# Exceptional points at bound states in the continuum in photonic integrated circuits

**DOI:** 10.1515/nanoph-2022-0420

**Published:** 2022-11-14

**Authors:** Haoye Qin, Xiaodong Shi, Haiyan Ou

**Affiliations:** Tsinghua Shenzhen International Graduate School, Tsinghua University, Shenzhen 518055, China; Laboratory of Wave Engineering, School of Electrical Engineering, EPFL, Lausanne, Switzerland; Department of Electrical and Photonics Engineering, Technical University of Denmark, Kgs. Lyngby 2800, Denmark

**Keywords:** bound states in the continuum, exceptional points, photonic integrated circuits

## Abstract

We propose the realization of exceptional points (EP) at bound states in the continuum (BIC), with two coupled strips, made of an electron-beam resist and patterned on the thin film photonic integrated platform, which makes possible etchless photonics integrated circuits (PIC). The loss rate of the EP can be significantly decreased through merging the BIC peaks in the dual-BIC scheme. The orthogonality of the eigenvectors is retrieved for evaluating the Hermitian orthogonal eigenvectors and the non-Hermitian EP features. We also find that engineering the dimension of the dual-BIC scheme enables a transition between the coalesced eigenvectors in the EP and the orthogonal eigenvectors in the Hermitian system. This work is of great significance for the exploration on BIC-based directional coupling with ultralow-loss phase matching conditions, special coupling conditions of EPs and BICs with coupled quasi-BIC systems, dynamical EP encircling, and EP topology, in PICs.

## 1 Introduction

Bound states in the continuum (BICs) are special wave solutions that are embedded in a radiative continuum, but remain localized without coupling to the extended waves or radiation [1]. Although the concept of BIC originates from quantum mechanism, various demonstrations have been recently reported in photonics [2], [3], [4], [5], [6], [7], [8]. The BIC induced light trapping has been realized by controlling the incident angle of light launching onto a photonic crystal slab, resulting in a tunable quality factor towards infinity [9]. BICs in isolated subwavelength nanoparticle resonators have been explored by employing the mode coupling between the Mie-like mode and the Fabry–Perot mode [10], [11], [12]. The in-plane symmetry and the broken symmetry in periodic structures lead to BICs, which significantly enhances the field localization, improves the optical chirality, and increases the efficiency of the nonlinear processes [13], [14], [15]. The formation of BICs in hybrid plasmonic–photonic systems has also been studied, where the lossy plasmonic mode is pushed towards the BIC with an increasing quality factor [16]. Recently, a novel type of BIC with fundamentally new photonic architecture has been demonstrated, through patterning a strip waveguide, made of a low-refractive-index material on a high-refractive-index material based photonic integrated platform, which can couple the transverse-electric (TE) mode in the continuum and the transverse-magnetic (TM) bound mode in the thin film [17], [18], [19]. The scheme combines the BICs with the photonic integrated circuits (PICs), showing great advantages. The fabrication processes are very simple, which only requires a single lithography step without etching, and there is no strip-waveguide sidewall roughness induced scattering, as the light propagates in the thin film. Hence, it could practically benefit the PICs made of the materials with high hardness or high chemical inertness, such as diamond, silicon carbide, and lithium niobate (LN), which are difficult to etch [20], [21], [22], [23].

Hermitian degeneracy can lead to the diabolic point (DP), and its eigenvalue splitting has a linear response to the external perturbation [24]. DPs have more practical feasibility in comparison with exceptional points (EPs), which typically require balanced gain and loss [25]. It is featured with a geometric phase and holds potential applications in topology and quantum information [25, 26]. The past few years have seen the development of non-Hermitian optics and the introduction of promising physical systems, which contribute to both theoretical and experimental demonstrations of parity-time (PT) symmetry, anti-PT symmetry, and EPs [24, 27], [28], [29], [30]. The EP is a singular point, where the real and the imaginary parts of the eigenvalues become coalesced, and it has been applied for enhanced sensing due to the square-root response to the external perturbation [31], [32], [33]. EPs can be approached by tailoring the detuning, gain–loss ratio, and coupling strength between two or more coupled components [34]. The eigenvectors at DPs show orthogonal features, and they become coalesced at EPs, making the system behave as if it loses the dimensionality [24]. It is noteworthy that the EP and DP at BICs have been mainly studied in flat optics, but have not been explored in optical planar waveguides in PICs.

In this work, we propose a novel method to obtain the EPs at BICs in PICs, for the first time to the best of our knowledge. A dual-BIC scheme is achieved by patterning two coupled low-refractive-index polymer waveguides on the LN photonic integrated platform, which couples TE continuum modes and TM bound modes. We investigate the interaction between the two BIC modes, propose a feasible method to tailor the dual BIC scheme, and achieve EPs with a low loss rate in PICs. The dual-BIC system also shows a transition behavior, enabling a transition from non-Hermitian EPs to the feature with orthogonal eigenstates in vicinity of a Hermitian DP. The proposed dual-BIC induced EPs can benefit directional coupling with ultralow-loss phase matching, the on-chip integration of low-loss EPs for enhanced sensing, all-optical modulation, and optical nonreciprocal transmission [24, 27, 34].

## 2 Results and discussions

**Figure 1:**
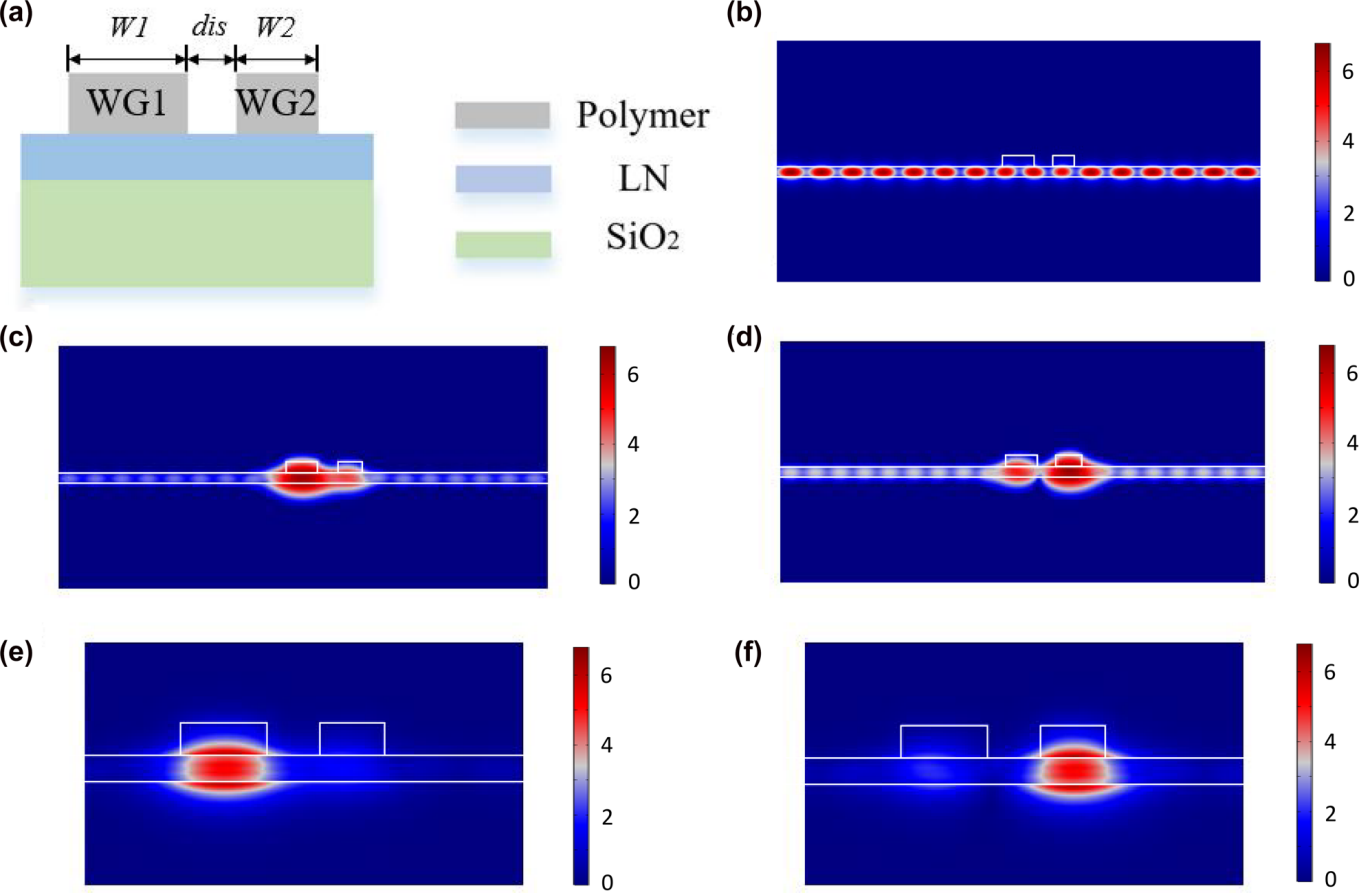
Coupled polymer strip waveguides based dual-BIC scheme. (a) Cross section of the structure. Normalized electric field distribution of (b) the TE continuous mode, (c) and (d) the TM leaky modes, and (e) and (f) the TM bound modes in the dual-BIC scheme. The TE eigenmode has dominant TE continuum mode that locates in the LN layer, and the TM one is mainly located under the polymer strip.

For a single polymer waveguide, by tuning the geometric parameters, the TM bound mode can be decoupled from the TE continuous modes, generating the BIC based on destructive interference between different coupling channels. For the coupled polymer waveguides, there exists one BIC in each waveguide and two BICs in total with zero coupling strength (infinite gap). When reducing the gap, the two BICs start to distort, and the loss rate is increased, due to mode coupling. However, they can still be accessed separately, and fine-tuned parameters can result in a merging BIC effect. Figure 1(a) shows the schematic of two coupled polymer (ZEP520, e-beam resist) based strip waveguides (WG1 and WG2) with a thickness of 500 nm on a 300 nm thick LN thin film, which can be directly patterned through a single e-beam lithography process. The following simulation is carried out in COMSOL Multiphysics. The refractive index of LN is set as *n*_o_ = 2.21 and *n*_e_ = 2.13, and the refractive index of the polymer is set as 1.54, at 1550 nm. When two polymer strip waveguides are parallel placed closely, the TE continuum modes exist, as shown in Figure 1(b). The existence of the TE continuum modes leads to a TM leaky mode, as shown in Figure 1(c) and (d). Under special circumstances, the coupling between the TE continuum modes and the TM bound modes can lead to well-confined BIC modes, shown in Figure 1(e) and (f).

**Figure 2:**
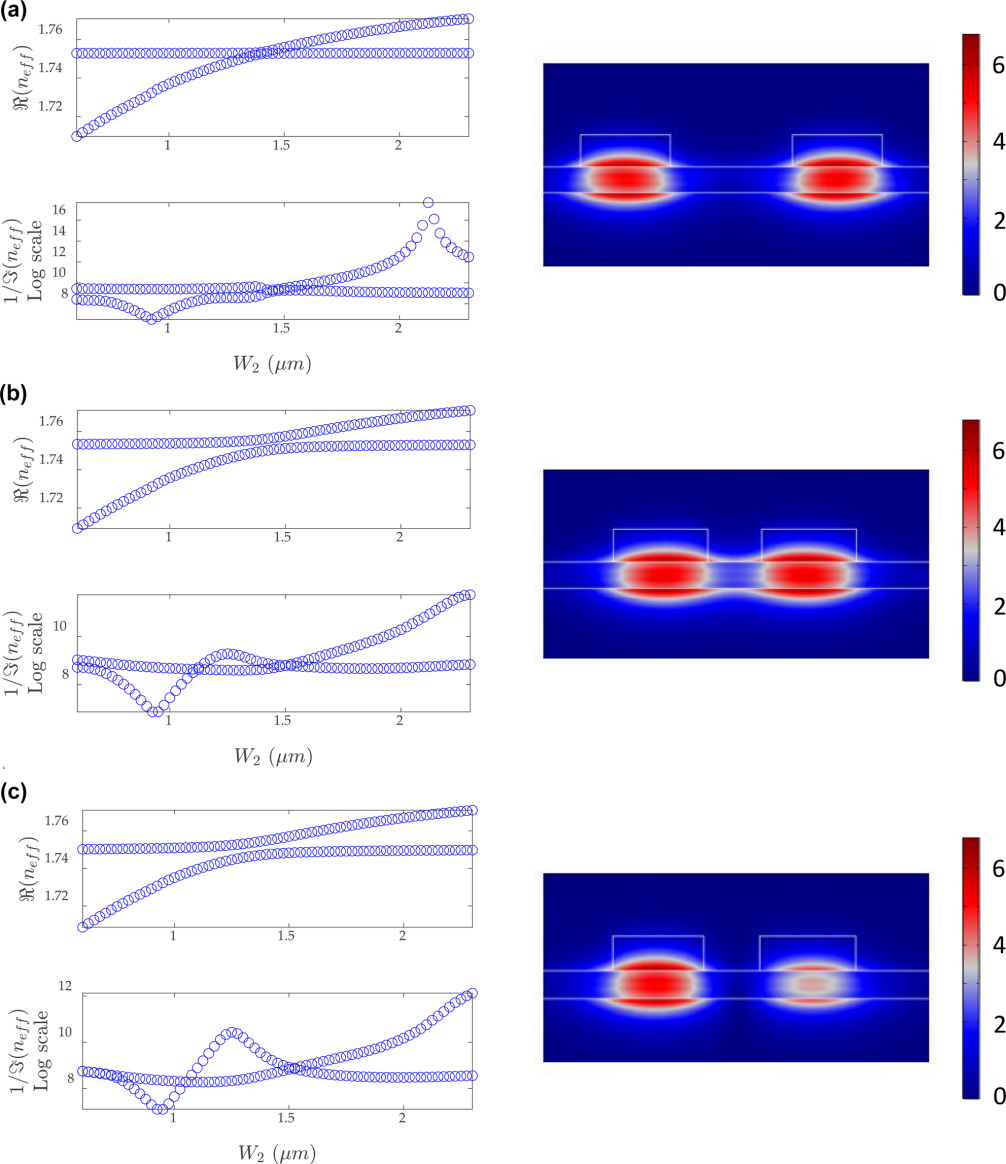
The real and the imaginary parts of *n*_eff_ of the dual modes versus *W*_2_ and the corresponding normalized electric field mode profile at or near the EP, with structural parameters of (a) *W*_1_ = 1.4 µm, dis = 1.9 µm, (b) *W*_1_ = 1.4 µm, dis = 0.8 µm, and (c) *W*_1_ = 1.3 µm, dis = 0.8 µm.

A distinctive feature resulting from the mode coupling is the EP, at which both the real and the imaginary parts of the eigenvalues become coalesced. The dual-BIC scheme can be employed to realize the EPs by modulating the mode profiles and the coupling strength between the coupled modes, through tailoring the widths of the strip waveguides (*W*_1_ for WG1 and *W*_2_ for WG2) and the gap distance (dis) between them. In Figure 2(a)–(c), *W*_2_ is swept from 0.6 µm to 2.5 µm, and the complex effective index, *n*_eff_, corresponding to complex eigenvalues in waveguides, is simulated with fixed *W*_1_ and dis. The imaginary part of *n*_eff_ is plot inversely in a logarithmic form, which is defined as the loss rate. Figure 2(a) shows *n*_eff_ versus *W*_2_, when *W*_1_ = 1.4 µm and dis = 1.9 µm. The real part of *n*_eff_ of the mode in WG1 is insensitive to *W*_2_, since dis is too large to induce strong coupling strength. However, the mode coupling effect can be revealed from the loss diagram, as the imaginary part of *n*_eff_ is extensively modulated. By increasing *W*_2_, the mode in WG1 becomes more lossy. While for the case in Figure 2(b) and (c), increasing *W*_2_ will not always lead to more loss for the mode in WG1. The evolution of loss rate in *W*_1_ or *W*_2_ is based on the coupling strength (waveguide gap) and eigenvalues of each waveguide (waveguide width). The dip and the peak in the loss diagram correspond to a super leaky mode and a BIC mode, respectively. Meanwhile, an EP is found, where the real and imaginary parts of *n*_eff_ of the two modes are equivalent simultaneously, indicating that the TM bound modes are equally excited. Figure 2(b) and (c) shows *n*_eff_ versus *W*_2_, with the same small dis = 0.8 µm but different *W*_1_ of 1.4 µm and 1.3 µm, respectively. The results demonstrate that the coupling strength is enhanced with a decreasing dis, and the real part of *n*_eff_ of the mode in WG1 can be modulated by changing *W*_2_, which results in a deviation from the EP. The mode profiles in the coupled waveguides become asymmetric, as the evanescent waves perturb the TE continuum mode distributing in the LN thin film, which leads to an interaction between the two bound modes. The mode coupling effect between the two evanescently coupled waveguides can be described by a 2 × 2 Hamiltonian [35, 36], given by

(1) $$\left[\begin{array}{cc} \beta_{1}-\text{i}\gamma_{1} & \kappa \\ \kappa & \beta_{2}-\text{i}\gamma_{2} \end{array}\right]\left[\begin{array}{c} \phi_{1}\\ \phi_{2} \end{array}\right]=E\left[\begin{array}{c} \phi_{1}\\ \phi_{2} \end{array}\right].$$

Here, *β*_1,2_ − i*γ*_1,2_ is the original eigenvalue of the two individual waveguides under the uncoupling condition, *E* is the eigenvalue under the coupling condition, *κ* is the coupling strength, and ${\left[\phi_{1}\;\phi_{2}\right]}^{\text{T}}$ is the eigenvector. Since we are focusing on coupled two quasi-BIC modes that are mainly located under the polymer waveguide, the dominant coupling will be the evanescent coupling between the two polymer waveguides. Therefore, we omit the coupling from TM bound modes to the TE continuum mode when analyzing the coupling between two TM bound modes.

**Figure 3:**
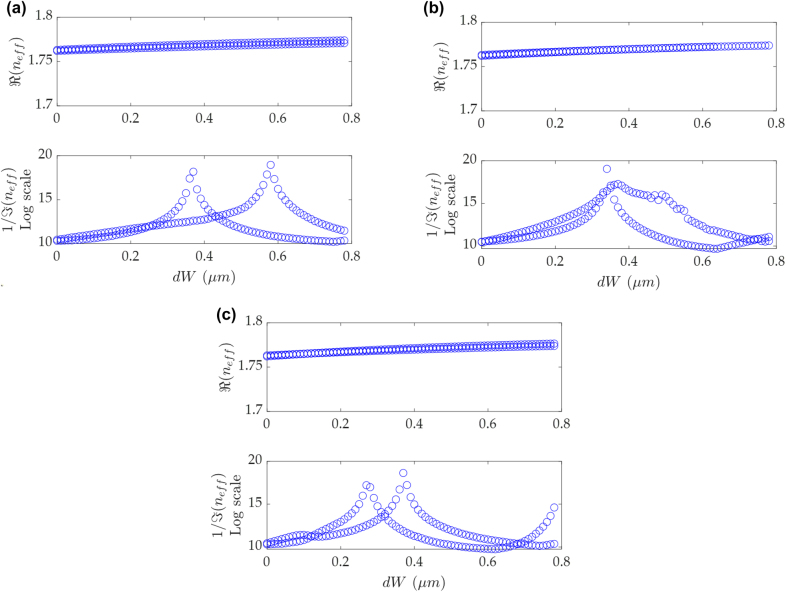
The real and the imaginary parts of *n*_eff_ of the dual modes versus d*W* with structural parameters of *W*_1_ = 1.8 µm, dis = 1.5 µm, and (a) *C* = 0.7, (b) *C* = 1.1, and (c) *C* = 1.5, to keep the equivalent real parts of *n*_eff_. The widths of two waveguides are $W_{1}^{\prime }=W_{1}+dW$ and $W_{2}^{\prime }=W_{2}+C\times dW$ , individually.

The two coupled bound modes are supposed to have different BIC conditions in the dual-BIC scheme. Therefore, we investigate the possibility of merging two BIC peaks in the dual-BIC scheme to reduce the loss rate at the EP, by changing *W*_1_ and *W*_2_ simultaneously, but with a different ratio of *C*, i.e. $W_{1}^{\prime }=W_{1}+dW$ and $W_{2}^{\prime }=W_{2}+C\times dW$ , in order to maintain the equivalent real part of *n*_eff_ of the two modes. Figure 3 shows that the imaginary part of *n*_eff_ is significantly modulated through this method. In Figure 3(a), each mode shows a peak in the loss diagram, and the crossing point between the two peaks indicates the formation of the EP, assisted by two quasi-BIC modes. By changing *C*, the BIC peak can be shifted to approach, overlap and depart from the other BIC peak, as shown in the loss diagrams. Figure 3(b) shows the maximums of the two imaginary parts occur at almost the same d*W*, when *C* = 1.1. Although the coupling effect in this dual-BIC scheme undermines one BIC lineshape to a quasi-BIC, it still makes possible an EP with much lower loss.

**Figure 4:**
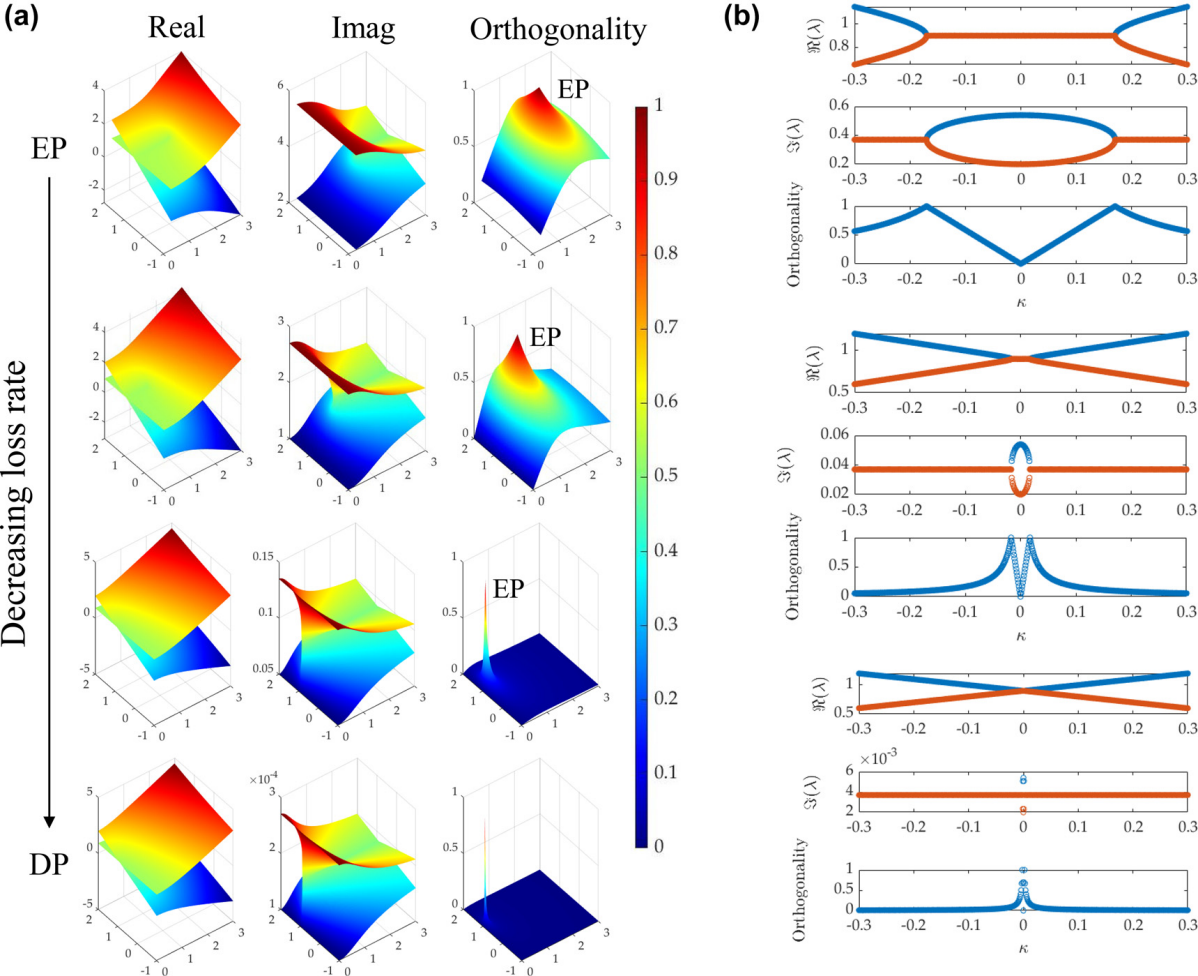
Evolution of EPs and their orthogonality with reducing loss rate. (a) Riemann surfaces of the real (first column) and the imaginary (second column) parts of eigenvalues cross at the EP and the orthogonality around the EP (third column) with a loss rate reduced by a factor of 1, 2, 20, and 500. The surfaces are obtained by sweeping frequency detuning and coupling strength with normalized values. (b) The real and the imaginary parts of the eigenvalues and the orthogonality versus the coupling strength with a loss rate reduced by a factor of 1, 4, and 20.

Since it is hard to distinguish EPs and the Hermitian feature in low-loss systems, in order to demonstrate the transition, the numerical calculation is conducted with a coupled two-component Hamiltonians. Figure 4(a) demonstrates the transition from EPs to the Hermitian feature by reducing the loss rate. For the real part of the eigenvalues, the two Riemann surfaces change from a cross with a line to a cross at a point. The third column shows the orthogonality of eigenvectors, defined as $v_{1}^{H}v_{2}/\left|v_{1}^{H}v_{2}\right|$ , where *H* is the Hermitian operator and *v*_1,2_ is the eigenvectors of the system. The normalized orthogonality should approach 1 for EPs with coalseced eigenvectors. The coalesced eigenvectors at EPs severely skew the vector spaces. With a high loss rate, two Riemann surfaces can be perceived more easily with a cross, and the orthogonality is non-zero in most of the region. Therefore, we use the orthogonality to verify and distinguish the EP (coalesced eigenvectors) and the Hermitian system (orthogonal eigenvectors) based on the fact that eigenvectors of distinct eigenvalues are orthogonal for a Hermitian matrix. With a low loss rate, it is hard to observe the cross of two surfaces, and the EP becomes a singular point in a sharp peak detached from the plane, indicating it is difficult to approach the EP with extremely low loss. Figure 4(b) shows the one-dimensional evolution of the eigenvalues (*λ*) and the orthogonality versus the coupling strength (*κ*) at a certain value of detuning with a decreasing loss rate. The splitting at the EP against the perturbation in the real part of the eigenvalue shows a square-root relation for high loss and a linear relation for low loss, closed to the DP [37]. It becomes difficult to distinguish EPs and Hermitian feature with low loss, as the orthogonality jumps rapidly from 1 to 0, when *κ* is approaching 0.

**Figure 5:**
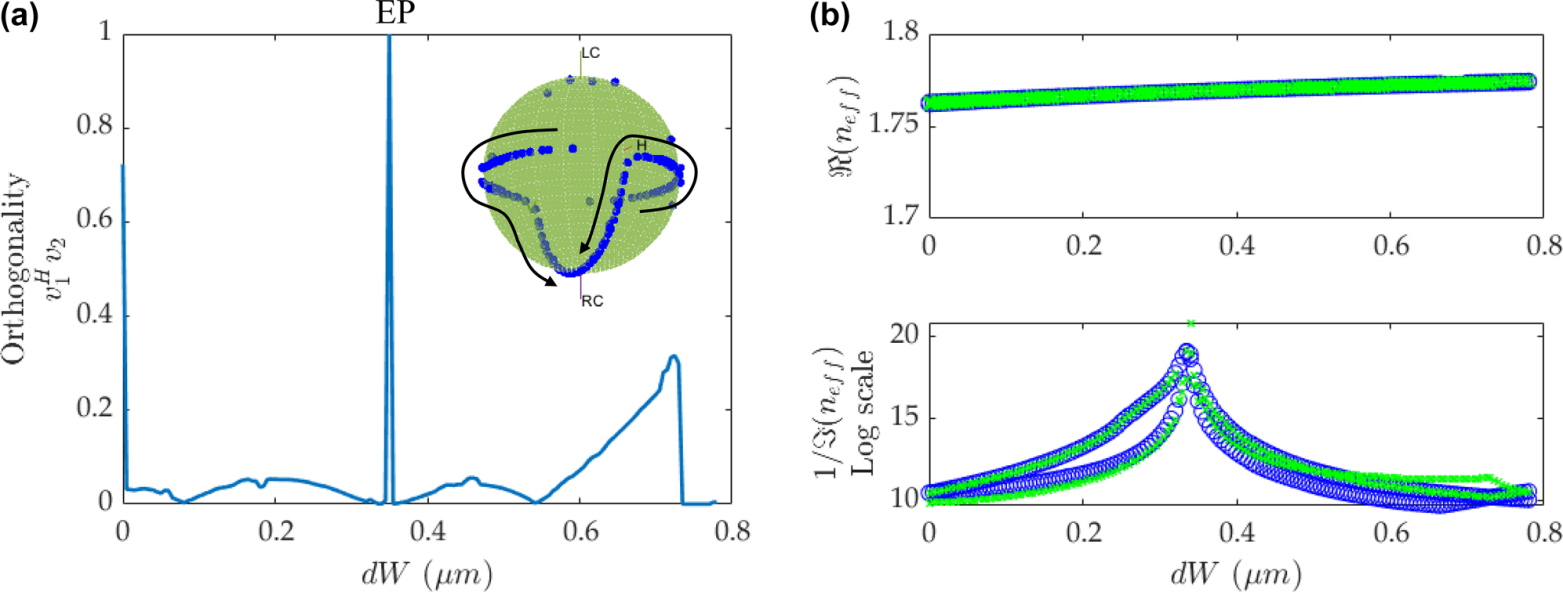
EPs at BICs. (a) The orthogonality versus d*W* with *C* = 1.17. Each two-row eigenvector can be deemed like a polarization state and mapped onto the Bloch sphere. At EP, with coalesced eigenstates, the two points will also coalesce on the Bloch sphere. The inset shows the corresponding evolution of the eigenvectors on the Bloch sphere. The imaginary part is magnified by a factor of 10^4^ for better visualization. The arrow shows the corresponding evolution of eigenstates on the Bloch sphere. (b) The real and imaginary parts of *n*_eff_ versus d*W*. The blue circles show the *n*_eff_, obtained from the direct eigenmode simulation, and the green crosses show the calculated eigenvalues, obtained from the Hamiltonian for the coupled waveguides.

To evaluate the features of EPs and Hermitian by the orthogonality when the system is pushed towards the EP at BICs, the BIC peaks in the loss diagram are forced to merge at the maximum point by tailoring *C*. When *C* is fine tuned to be 1.17, the two BIC peaks in the loss diagram overlap at their maximum points, seen in Figure 5(b), which indicates the EP at BIC is obtained in the merging dual-BIC scheme. The blue circles are *n*_eff_ obtained from the eigenmode simulation, and the green crosses are the calculated eigenvalues, solved from the Hamiltonian for the coupled waveguides. The uncoupled eigenvalues (*β*_1,2_ and *γ*_1,2_) are simulated by placing each individual waveguide alone, and extracted from the real and imaginary parts of the effective index. With the original eigenvalues and *E*_1,2_, the coupling strength can be obtained with fitting, so that the eigenvectors for the Hamiltonian can be calculated. The calculated eigenvalues from the Hamiltonian agree well with the simulated eigenvalues.

The orthogonality versus d*W* is plotted in Figure 5(a), and there are three peaks tending to have non-zero value of orthogonality in the coupled waveguide scheme with different widths. At d*W* = 0.37 µm, the orthogonality is unity, which indicates the EP is exactly at BICs. The other two peaks are induced by the crossing points in the loss diagram at d*W* = 0.02 µm and 0.73 µm. Since the values of orthogonality are not unity, their eigenstates are close to EP but not exactly coalesced. Since the eigenvectors completely coalesce at an EP, the orthogonality parameters should approach 1 for all the EPs and not just the EPs at BICs. While the BIC feature can be identified from the peak in the loss diagram. For the EP at BICs, the orthogonality shows a very sharp peak, revealing the extreme sensitivity to the parameters and making the EP very hard to perceive, which agrees well with the analysis in Figure 4. It is noticed that the merging of BICs to obtain EPs with low-loss rate is achieved by modulating of the coupled modes in the dual-BIC scheme rather than the independent BICs under two waveguides, due to the specifically asymmetric geometry of the waveguides and the different loss evolution of the coupling between two eigenmodes.

The eigenvectors are illustrated on the Bloch sphere in the inset of Figure 5(a). The points located on the equator represent the orthogonal eigenvectors with the Hermitian feature, when d*W* is away from the EP at BICs. The eigenvectors move symmetrically towards two poles, when d*W* approaches the EP at BICs. The exact EP appears, when the two eigenvectors coalesce at the north/south pole. Two BIC modes with ultralow-loss appear at nearly the same position, indicating an EP is pushed towards the BIC. It is noteworthy that the ideal BIC cannot be achieved, but it can become quasi-BIC with extremely low loss rate approaching BIC, as revealed from the peaks in the loss diagram. The strategy of making the two BIC peaks overlap is similar to the merging BICs illustrated in Ref. [38], where a BIC pair is tuned toward merging for a broad wave vector range and constructing merging BICs at an arbitrary point in the reciprocal space. In comparison, the merging of BICs in this work, based on the coupled modes in waveguides, can generate the low-loss state of the EPs and have the possibility of pushing the EP towards the BICs.

**Figure 6:**
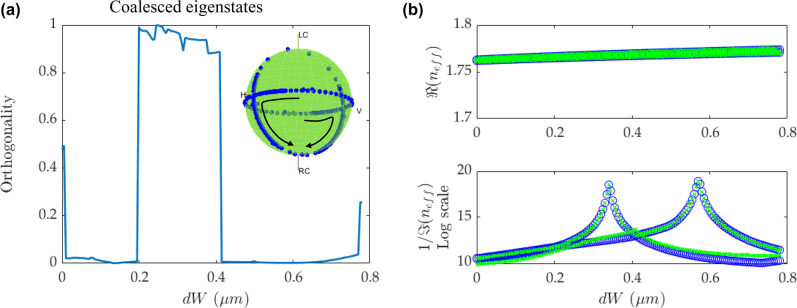
Transition between coalesced eigenstates and orthogonal eigenstates under extremely low-loss condition. (a) The orthogonality versus d*W* with *C* = 0.7. The inset shows the corresponding evolution of the eigenvectors on the Bloch sphere. The imaginary part is magnified by a factor of 10^4^ for better visualization. The arrow shows the corresponding evolution of eigenstates on the Bloch sphere. (b) The real and imaginary parts of *n*_eff_ versus d*W*.

Figure 6 shows the case of separated BIC peaks with *C* = 0.7 in the loss diagram. Figure 6(a) shows a broad range of the orthogonality near the unity. The two transition points correspond to the intersection points in the loss diagram in Figure 6(b). The jump of the orthogonality in Figure 6(a) from near 1 to 0 after d*W* = 0.4 µm corresponds to the crossing point in the loss diagram in Figure 6(b). In the range of d*W* = 0.2–0.4 µm, one of the modes has a peak in the loss diagram (quasi-BIC), and the coupled waveguide scheme tends to reveal the feature of nearly coalesced eigenstates in this range. However, there are not always coalesced eigenvalues in this range, as shown in Figure 6(b). It is because when the two BIC peaks are splitted in contrast to the dully-tuned case in Figure 5, d*W* mostly affects only one mode, and its eigenstate is not as sensitive as the loss diagram under the near-zero loss condition. In this range, it is not exactly EP with unequal loss rate, but since the loss diagram is illustrated in logarithmic scale, the discrepancy is difficult to observe. After d*W* increases beyond 0.4 µm, it mainly plays a role on the other mode, leading to a second BIC peak. This mode shift eliminates the coalesced eigenstates and generates near-zero orthogonality. In the range of d*W* = 0.4–0.65 µm, the feature of the Hermitian system with orthogonal eigenvectors is expected, and along with the equivalent real eigenvalues, it approaches the degeneracy point DP, as the absolute values of loss rate are extremely low, close to zero, and thus can be negligible. Therefore, the system can provide an effective way to evaluate the transition from the non-Hermitian EP to the Hermitian system with orthogonal eigenstates approaching a DP.

## 3 Conclusions

In conclusion, we propose a method to obtain the ultralow loss EPs in PICs through a dual-BIC scheme with coupled polymer waveguides. By tailoring the width of the two coupled polymer waveguides on the LN thin film, two BICs can be merged to obtain EPs at BICs. We also explore the effect of the loss rate on the orthogonality of the eigenvectors to study the behaviors of EPs and DPs, and demonstrate the capability of transition from the non-Hermitian EP to the feature similar to a Hermitian DP with orthogonal eigenstates. This work paves way for further exploration on BIC-based directional coupling with ultra-low-loss phase matching conditions, as well as special coupling conditions of EPs and BICs with coupled quasi-BIC systems, dynamical EP encircling, and EP topology, in PICs.
